# A Neural Mass Model to Simulate Different Rhythms in a Cortical Region

**DOI:** 10.1155/2010/456140

**Published:** 2009-12-01

**Authors:** M. Zavaglia, F. Cona, M. Ursino

**Affiliations:** Department of Electronics, Computer Science, and Systems, University of Bologna, Via Venezia 52, 47023 Cesena, Italy

## Abstract

An original neural mass model of a cortical region has been used to investigate the origin of EEG rhythms. The model consists of four interconnected neural populations: pyramidal cells, excitatory interneurons and inhibitory interneurons with slow and fast synaptic kinetics, GABA_A, slow_ and GABA_A,fast_ respectively. A new aspect, not present in previous versions, consists in the inclusion of a self-loop among GABA_A,fast_ interneurons. The connectivity parameters among neural populations have been changed in order to reproduce different EEG rhythms. Moreover, two cortical regions have been connected by using different typologies of long range connections. Results show that the model of a single cortical region is able to simulate the occurrence of multiple power spectral density (PSD) peaks; in particular the new inhibitory loop seems to have a critical role in the activation in gamma (*γ*) band, in agreement with experimental studies. Moreover the effect of different kinds of connections between two regions has been investigated, suggesting that long range connections toward GABA_A,fast_ interneurons have a major impact than connections toward pyramidal cells. The model can be of value to gain a deeper insight into mechanisms involved in the generation of *γ* rhythms and to provide better understanding of cortical EEG spectra.

## 1. Introduction

Neuronal activity in the *γ* band has been proposed as a physiological indicator of perceptual and higher cognitive processes in the brain, including arousal and attention [1], binding of stimulus features and perception of objects [2], consciousness, and language [3]. Generally, *γ* rhythms are found in EEG spectra together with other rhythms, such as locally generated activity in the beta (*β*) band or thalamocortical activity in the alpha (*α*) band.

In this context, mathematical models can be of the greatest value to gain a deeper insight into the mechanisms involved in the generation of *γ* rhythms and to provide better understanding of cortical EEG spectra. Models in fact can mimic electrical activity of groups of neurons, taking into account different patterns of connectivity, and simulate brain electrical activity in regions of interest.

In recent years many authors used neural mass models to study the generation of EEG rhythms. In these models the dynamics of entire cortical regions is generally represented with a few state variables, which mimic the interaction among excitatory and inhibitory populations, arranged in a feedback loop. In particular, neural mass-models of cortical columns, particularly useful to simulate some aspects of EEG signals, were developed by Lopes da Silva et al. [4] and by Freeman [5] in the late seventies, and subsequently improved and extended by Jansen and Rit [6] and Wendling et al. [7].

An intrinsic limitation of these models, however, consists in the way *γ* rhythms can be generated. Basically, one can obtain a *γ* rhythm as a consequence of the interaction between pyramidal neurons and fast inhibitory interneurons, provided that small values are used for the synaptic time constants [8]. Conversely, some recent works, both experimental and computational [9, 10], suggest that *γ* rhythms can be generated by a chain of fast inhibitory interneurons, even without the participation of other types of neurons. A further limitation of previous mass models consists in the difficulty to obtain multiple rhythms within the same cortical region, especially in the *β* and *γ* bands. To this end, in previous works at least two regions with different synaptic kinetics have been used to obtain the presence of two peaks in PSD [11].

The present work was devised, within the framework of neural mass models, to overcome the previous limitations. In particular two main objectives have been pursued: (i) to enrich the model of a single cortical region with a new feedback loop, through which fast inhibitory interneurons can produce a *γ* rhythm per se (i.e., without the participation of the other neural populations), and (ii) to demonstrate that the modified model can easily produce EEGs PSD of a single cortical region characterized by several peaks (i.e., several activities in different bands), using a very parsimonious description of connectivity weights.

The model is first presented in a synthetic form. Then, the role of connectivity between populations of excitatory and inhibitory interneurons internal to the cortical region is studied, with particular attention to the role of GABA_A,fast_ interneurons in the generation of *γ* activity. Subsequently, the effect of connectivity between two cortical regions is simulated. The discussion underlines the main virtues and limitations of the proposed model and points out the main aspects for future research.

## 2. Material and Methods

### 2.1. Model of a Single Cortical Region

**Figure 1:**
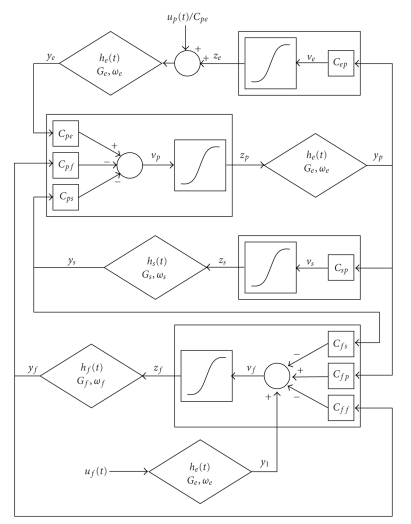
Model layout: four neural populations (pyramidal cells, excitatory interneurons, GABA_A,slow_ inhibitory interneurons, and GABA_A,fast_ inhibitory interneurons) which communicate via excitatory and inhibitory synapses. Worth noting is the presence of a new feedback loop with gain *C*_*f f*_.

The model of a cortical region presented here is a modified version of the model proposed by Wendling et al. [7]. It consists of four neural populations which communicate via excitatory and inhibitory synapses: pyramidal cells, excitatory interneurons, inhibitory interneurons with slow synaptic kinetics (GABA_A,slow_), and inhibitory interneurons with faster synaptic kinetics (GABA_A,fast_). In the following, a quantity which belongs to a neural population will be denoted with the subscripts *p* (pyramidal), *e* (excitatory interneuron), *s* (slow inhibitory interneuron), and *f* (fast inhibitory interneuron). Each neural population receives an average postsynaptic membrane potential from the other populations and converts the average membrane potential into an average density of spikes fired by the neurons. Three different kinds of synapses are used to describe the synaptic effect of excitatory neurons (both pyramidal cells and excitatory interneurons), of slow inhibitory interneurons and of fast inhibitory interneurons. Each synapse is simulated by an average gain (*G*_*e*_, *G*_*s*_, *G*_*f*_ for the excitatory, slow inhibitory and fast inhibitory synapses, resp.) and a time constant (in the model, the reciprocal of these time constants is denoted as *ω*_*e*_, *ω*_*s*_, and *ω*_*f*_ *, *resp.). The average numbers of synaptic contacts among neural populations are represented by eight variables (*C*_*i j*_). The inputs to the model (*u*_*p*_(*t*)*  *and* u*_*f*_(*t*)) excite pyramidal neurons and fast inhibitory interneurons, respectively, and represent all exogenous contributions, both excitation coming from external sources and the density of action potentials coming from other regions. Inputs to the other two populations have only a scanty effect on model dynamics, and hence have been neglected. The output of the model is represented by the membrane potential of pyramidal cells. Compared with the model described in our previous work [8], the new model exhibits two changes (see Figure 1): (i) fast inhibitory interneurons may receive an external input (say *u*_*f*_(*t*)) from pyramidal neurons of other regions; (ii) fast inhibitory interneurons exhibit a negative self-loop; that is, they not only inhibit pyramidal neurons (as in previous model) but also inhibit themselves. This idea agrees with the observation that basket cells in the hippocampus and cortex are highly interconnected and a chain of fast inhibitory interneurons can induce *γ* activity per se (i.e., even without the participation of other neural populations) thanks to its internal self-inhibitory connections [9].

### 2.2. Model of Connectivity among Regions

In order to study how the cortical regions interact, we then considered a model composed of two cortical regions which are interconnected through long-range excitatory connections. In the following the superscript *k* will be used to denote a presynaptic region and the superscript *h* to denote the target (post-synaptic) region. To simulate connectivity, we assumed that the average spike density of pyramidal neurons in the pre-synaptic region (*z*_*p*_^*k*^) affects the target region via a weight factor, *W*_*j*_^*hk*^ (where *j* = *p* or *f*, depending on whether the synapse targets to pyramidal neurons or fast inhibitory interneurons), and a time delay, *T* (assumed equal for all synapses). This is achieved by modifying the input quantities *u*_*p*_^*h*^(*t*) and/or *u*_*f*_^*h*^(*t*) as follows:

(1) $$\begin{array}{c} u_{j}^{h}\left(t\right)=n_{j}^{h}\left(t\right)+W_{j}^{hk}z_{p}^{k}\left(t-T\right), \end{array}$$

where *n*_*j*_(*t*) represents a Gaussian white noise (in the present work: mean value *m*_*j*_ = 0 and variance *σ*_*j*_^2^ = 5).

## 3. Results

### 3.1. Sensitivity Analysis on a Single Cortical Region

The simulations performed in a previous paper [8] demonstrate that a model of a single cortical region, stimulated with input white noise to pyramidal cells (*u*_*p*_(*t*)), produces just a unimodal spectrum (i.e., a spectrum with a single well defined peak) whose position primarily depends on the synaptic kinetics (i.e., *ω*_*e*_, *ω*_*s*_, *ω*_*f*_) parameters. In order to obtain multiple rhythms in the spectrum, this model needs the contribution of other rhythmic external sources that induce their activity on it.

**Figure 2:**
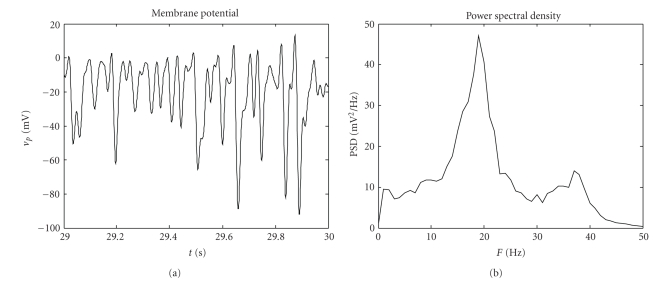
Output of the model of a single cortical region in terms of membrane potential (a) and PSD (b), simulated with the basal parameters reported in Table 1.

The most interesting feature presented here is the ability of generating more than one oscillatory rhythm within a single region. Figure 2 shows the membrane potential and the corresponding PSD obtained with the model of a single cortical region simulated with the basal parameters reported in Table 1. It is worth noting that this model produces a bimodal spectrum, with two intrinsic and well-defined peaks, oscillating, respectively, in *β* and *γ* ranges. Differently from previous works [7, 8], in these simulations we used a value for the time constant of GABA_A,fast_ interneurons of 17 milliseconds, in better agreement with [9].

**Figure 3:**
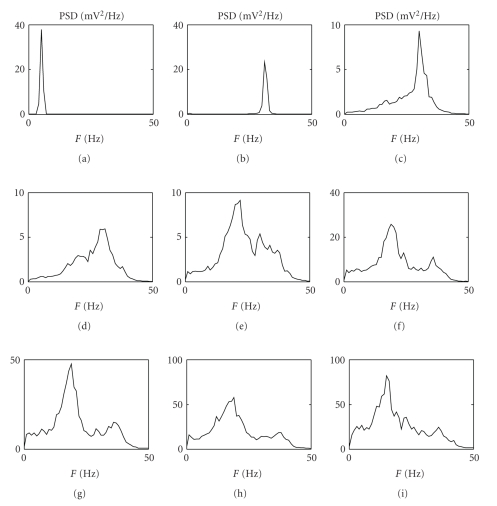
PSD of a single region obtained by varying the connection from GABA_A,fast_ interneurons to pyramidal cells (parameter *C*_*pf*_). The values of *C*_*pf*_ are 0C, 0.8C, 1.3C, 1.5C, 2C, 3C, 4C, 5C, and 6C.

Figure 3 shows the role of the GABA_A,fast_ loop in the generation of a second rhythm in the *γ* band. Each panel shows how the output of the model, in terms of PSD, changes by incrementing the connection *C*_*pf*_ from GABA_A,fast_ interneurons toward pyramidal cells. With the values reported in Table 1, but setting the value of *C*_*pf*_ to 0, the region exhibits a single rhythm around 5 Hz (first panel). Using small values of *C*_*pf*_ (0.8C and 1.3C) the region exhibits a single rhythm around 30 Hz (second and third panels, resp.). As *C*_*pf*_ increases (from 1.5C to 6C; see Figure 3 caption) the region exhibits two different well-evident rhythms, one in the *β* band and one in the *γ* band (fourth to ninth panels).

**Figure 4:**
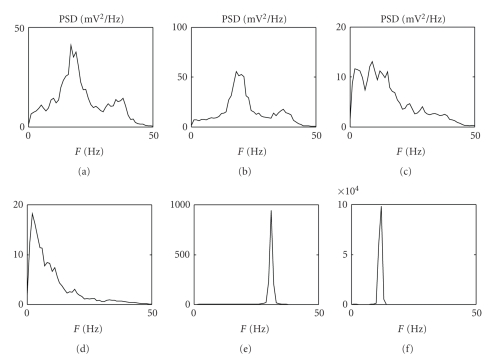
PSD of a single cortical region setting off some connections among neural populations. The first panel represents the output of the whole model with parameters as in Table 1. The other five panels represent the power spectra, respectively, when *C*_*ep*_ = 0, *C*_*sp*_ = 0, *C*_*f p*_ = 0, *C*_*fs*_ = 0, and* C*_*f f*_ = 0.

Figure 4 shows the results of a sensitivity analysis performed on the parameters of the model describing the loops among neural populations. The aim of this analysis is to identify the loops that are essential in order to obtain *γ* rhythms. In order to do this analysis five crucial connections are cut one at a time. The first panel shows the PSD of the region when all the connections among the neural populations are active (parameters as in Table 1). When connections from pyramidal cells toward excitatory interneurons or from GABA_A,slow_ interneurons toward pyramidal cells are set to 0 (second and third panels), the two rhythms persist. In the fourth panel the connection from pyramidal cells toward GABA_A,fast_ interneurons is set to 0. Even if two rhythms are not clearly distinguishable, the power band is still fairly broad (0–40 Hz). It is worth noting that the two rhythms collapse in a single one if the connections from GABA_A,slow_ interneurons toward GABA_A,fast_ interneurons or from GABA_A,fast_ interneurons toward themselves are cut (last two panels) suggesting a crucial role for these connections in the generation of a bimodal spectrum, composed of *β* and *γ* rhythms. It is worth noting that, in the absence of the loop among fast inhibitory interneurons (*C*_*f f*_ = 0), the model, with the assigned time constants, produces a rhythm in the alpha band.

### 3.2. Connectivity between Two Cortical Regions

**Figure 5:**
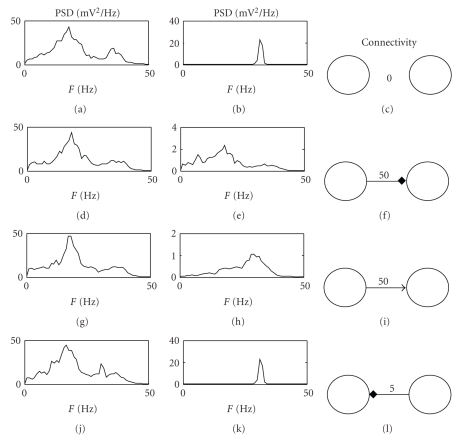
PSD of two regions (first region in the first column and second region in the second column) communicating by different connectivity patterns. The connectivity patterns are represented in the third column; the arrow indicates connectivity toward pyramidal cells; the square indicates connectivity toward GABA_A,fast_ interneurons. The first region is simulated using the parameters reported in Table 1, while the second region is obtained by modifying only *C*_*pf*_(*C*_*pf*_ = 0.08C).

Figure 5 shows the behaviour of a model composed of two interconnected regions. The first line of panels shows the PSD of the two regions when they are not connected to each other. Parameter *C*_*pf*_ is set to 4*C* in the first region, whereas *C*_*pf*_ is set to 0.8*C* in the second one. All other parameters have the same values as in Table 1. In this way, the first region exhibits two rhythms, and the second only one rhythm but at a different frequency. The second region can be forced to exhibit the same two rhythms as the first by introducing a connection toward GABA_A,fast_ interneurons (second line). A different spectrum, with a wide activity at high frequencies, is obtained if connectivity is directed toward pyramidal neurons (third line). A small connectivity from the second region towards GABA_A,fast_ cells of the first one makes the first region exhibit three simultaneous rhythms: the two intrinsic rhythms and a third acquired external rhythm (fourth line).

## 4. Discussion

In this work we used a neural mass model to study the presence of multiple rhythms in the EEG. The model presented here is modified compared with that used in our previous papers [8, 12]. The modifications aim at accentuating the role of the GABA_A,fast_ interneurons in the generation of rhythms in the *γ* band and obtaining a complex spectrum within a single cortical region. In order to study these aspects, we modified the model by adding a new input to the GABA_A,fast_ interneurons, and we introduced a feedback loop from the GABA_A,fast_ interneurons toward themselves (parameter *C*_*f f*_).

A sensitivity analysis on model parameters representing the internal connectivity among the four neural populations (see Figures 3 and 4) shows that strong short-range connections between GABA_A,fast_ interneurons and pyramidal cells and between GABA_A,fast_ interneurons and GABA_A,slow_ interneurons are fundamental for the generation of multiple rhythms in the EEG, and, above all, for the presence of a peak in the *γ* band. These results together confirm the findings, obtained in previous studies [9], that networks of fast inhibitory interneurons may be responsible for gamma activity in the brain. Another important result is represented by the use of more biologically plausible values for the time constants. In particular, the simulations performed with the modified model show that, contrarily to previous works [7, 8], one does not need to use very small values (a few ms) for the time constant of GABA_A,fast_ interneurons but more physiolgical values (13–20 ms) are able to induce oscillations at high frequency [9]. Another remarkable result is that, changing connections weights between populations, the rhythmic activity in the model can be easily moved between the alpha, beta, and gamma bands. This is a plausible physiological mechanism, since connectivity strength may be rapidly adjusted in vivo by synaptic plasticity. Hence, one does not need to hypothesize a less justifiable modulation of synaptic time constants to mimic the variety of rhythms encountered in vivo during motor and cognitive tasks. 

Finally, we studied the effect of the long range connections between two neural regions (see Figure 5). The simulations show that, using identical connection strengths (second and third line), the effect on the PSD is completely different, depending on the target population (GABA_A,fast_ interneurons or pyramidal cells). This original result emphasizes the predominant role of GABA_A,fast_ interneurons compared to pyramidal cells not only in generating multiple rhythms but also in rhythm transmission from one region to another. 

An important possible application of the present model consists in the estimation of effective connectivity between ROIs, starting from real EEG or MEG data. Actually, connectivity in the neurophysiological literature is commonly estimated using signal-based approaches (i.e., empirical black-box models). A major advantage in the use of interpretative models, instead of black-box models, lies in the physiological meaning of estimated parameters. Each parameter in an interpretative model possesses a clear physiological meaning and summarizes a specific mechanism involved in rhythm generation. Furthermore, interpretative models may permit a fusion between different techniques (e.g., EEG/MEG data and metabolic neuroimaging (fMRI or PET) data), since they allow different quantities (electrical and metabolic) to be simulated. Finally, the present model includes nonlinearitites (in particular sigmoidal relationships to describe neuron response) which are known to play a pivotal role in neural signal generation. By contrast, signal-based approaches generally assume the existence on linear relationships among signals. 

An actual problem in the use of neural mass models to estimate effective connectivity may be found in the excessive number of estimated parameters, especially when a large number of populations are simultaneously interacting. Estimation of many parameters may demand excessive computational resources and, above all, may lead to the problem of “overfitting” (i.e., good reproducibility of data but with little or none prediction capacity). Two considerations, however, may be followed to limit the number of estimated parameters in a neural mass model. First, not all the parameters in an ROI require estimation, but only a subset of them. In particular, we have shown that estimation of a single parameter for each region (*C*_*pf*_) may allow different rhythms to be produced and controlled quite finely. Second, parameters may be constrained by a priori knowledge (e.g., neuroanatomical data can be used to limit attention only to a few connectivity parameters, and knowledge on synaptic dynamic can be used to provide physiological values for time constants). Nevertheless, the problem of connectivity estimation using neural mass models is still at an initial stage and requires further theoretical and computational active research.

In conclusion, the results underline the performance of the model in the generation of multiple rhythms by using a simple connectivity circuit and may open new perspectives for the study of complex neural oscillations induced by the connection and synchronization among cortical regions. To our knowledge this is a first attempt to use a single neural mass model to reproduce a complex spectrum and investigate the role of fast inhibitory interneurons systematically. In perspective the model could be used as a simulator of the activity in brain regions involved in different cognitive tasks, and for testing the consequences of different connectivity patterns on EEG.

## Figures and Tables

**Table 1:** Model basal parameters.

*C* = 135	*C*_*sp*_ = 0.4C	*C*_*fs*_ = 0.2C	*G*_*e*_ = 5.17 mV	*ω*_*e*_ = 75s^−1^

*C*_*ep*_ = 0.4C	*C*_*ps*_ = 0.5C	*C*_*pf*_ = 4C	*G*_*s*_ = 4.45 mV	*ω*_*s*_ = 30 s^−1^

*C*_*pe*_ = 0.4C	*C*_*fp*_ = 0.4C	*C*_*ff*_ = 0.2C	*G*_*f*_ = 57.1 mV	*ω*_*f*_ = 60 s^−1^

## Appendix

The final model displayed in Figure 1 corresponds to the following set of differential equations:

Pyramidal neurons are

(A.1) $$\begin{array}{cc} \frac{dy_{p}\left(t\right)}{dt} & =x_{p}\left(t\right),\\ \frac{dx_{p}\left(t\right)}{dt} & =G_{e}\omega_{e}z_{p}\left(t\right)-2\omega_{e}x_{p}\left(t\right)-\omega_{e}^{2}y_{p}\left(t\right),\\ z_{p}\left(t\right) & =\frac{2e_{0}}{1+e^{-rv_{p}}}-e_{0},\\ v_{p}\left(t\right) & =C_{pe}y_{e}\left(t\right)-C_{ps}y_{s}\left(t\right)-C_{pf}y_{f}\left(t\right). \end{array}$$

Excitatory interneurons are

(A.2) $$\begin{array}{cc} \frac{dy_{e}\left(t\right)}{dt} & =x_{e}\left(t\right),\\ \frac{dx_{e}\left(t\right)}{dt} & =G_{e}\omega_{e}\left(z_{e}\left(t\right)+\frac{u_{p}\left(t\right)}{C_{pe}}\right)-2\omega_{e}x_{e}\left(t\right)-\omega_{e}^{2}y_{e}\left(t\right),\\ z_{e}\left(t\right) & =\frac{2e_{0}}{1+e^{-rv_{e}}}-e_{0},\\ v_{e}\left(t\right) & =C_{ep}y_{p}\left(t\right). \end{array}$$

Slow inhibitory interneurons are

(A.3) $$\begin{array}{cc} \frac{dy_{s}\left(t\right)}{dt} & =x_{s}\left(t\right),\\ \frac{dx_{s}\left(t\right)}{dt} & =G_{s}\omega_{s}z_{s}\left(t\right)-2\omega_{s}x_{s}\left(t\right)-\omega_{s}^{2}y_{s}\left(t\right),\\ z_{s}\left(t\right) & =\frac{2e_{0}}{1+e^{-rv_{s}}}-e_{0},\\ v_{s}\left(t\right) & =C_{sp}y_{p}\left(t\right). \end{array}$$

Fast inhibitory interneurons are

(A.4) $$\begin{array}{cc} \frac{dy_{f}\left(t\right)}{dt} & =x_{f}\left(t\right),\\ \frac{dx_{f}\left(t\right)}{dt} & =G_{f}\omega_{f}z_{f}\left(t\right)-2\omega_{f}x_{f}\left(t\right)-\omega_{f}^{2}y_{f}\left(t\right),\\ \frac{dy_{l}\left(t\right)}{dt} & =x_{l}\left(t\right),\\ \frac{dx_{l}\left(t\right)}{dt} & =G_{e}\omega_{e}u_{f}\left(t\right)-2\omega_{e}x_{l}\left(t\right)-\omega_{e}^{2}y_{l}\left(t\right),\\ z_{f}\left(t\right) & =\frac{2e_{0}}{1+e^{-rv_{f}}}-e_{0},\\ v_{f}\left(t\right) & =C_{fp}y_{p}\left(t\right)-C_{fs}y_{s}\left(t\right)-C_{ff}y_{f}\left(t\right)+y_{l}\left(t\right), \end{array}$$

where the sigmoid saturation is *e*_0_ = 2.5 s^−1^ and the sigmoid steepness is *r* = 0.56 mV^−1^. It is worth noting that we used a sigmoidal function centered in zero, which corresponds to the assumption that neurons normally work in the linear region.
